# Room Temperature Resonant Photocurrent in an Erbium Low-Doped Silicon Transistor at Telecom Wavelength

**DOI:** 10.3390/nano9030416

**Published:** 2019-03-11

**Authors:** Michele Celebrano, Lavinia Ghirardini, Marco Finazzi, Giorgio Ferrari, Yuki Chiba, Ayman Abdelghafar, Maasa Yano, Takahiro Shinada, Takashi Tanii, Enrico Prati

**Affiliations:** 1 Dipartimento di Fisica, Politecnico di Milano, Piazza Leonardo da Vinci 32, I-20133 Milano, Italy; michele.celebrano@polimi.it (M.C.); lavinia.ghirardini@polimi.it (L.G.); marco.finazzi@polimi.it (M.F.); 2 Dipartimento di Elettronica, Informazione e Bioingegneria, Politecnico di Milano, Via Colombo 81, I-20133 Milano, Italy; giorgio.ferrari@polimi.it; 3 School of Science and Engineering, Waseda University, 3-4-1 Ohkubo, Shinjuku, Tokyo 169-8555, Japan; chiba@tanii.nano.waseda.ac.jp (Y.C.); aymanabdelghafar@toki.waseda.jp (A.A.); m.ntr0504@ruri.waseda.jp (M.Y.); tanii@waseda.jp (T.T.); 4 Center for Innovative Integrated Electronic Systems, Tohoku University, Sendai 980-8572, Japan; shinada@cies.tohoku.ac.jp; 5 Istituto di Fotonica e Nanotecnologie, Consiglio Nazionale delle Ricerche, Piazza Leonardo da Vinci 32, I-20133 Milano, Italy

**Keywords:** erbium, silicon transistor, photocurrent

## Abstract

An erbium-doped silicon transistor prepared by ion implantation and co-doped with oxygen is investigated by photocurrent generation in the telecommunication range. The photocurrent is explored at room temperature as a function of the wavelength by using a supercontinuum laser source working in the μW range. The 1-μm^2^ transistor is tuned to involve in the transport only those electrons lying in the Er-O states. The spectrally resolved photocurrent is characterized by the typical absorption line of erbium and the linear dependence of the signal over the impinging power demonstrates that the Er-doped transistor is operating far from saturation. The relatively small number of estimated photoexcited atoms (≈$4\times 10^{4}$) makes Er-dpoed silicon potentially suitable for designing resonance-based frequency selective single photon detectors at 1550 nm.

## 1. Introduction

Er implanted in silicon has received a renewed interest after the advent of single-photon-based quantum communications, because of its capability to transmit [1,2] and receive [3] photons at a wavelength compatible with commercial optical fibers, and because of its compatibility with silicon photonics [4,5,6]. Er-doped silicon junctions [7,8] and transistors [3,9] have been explored in the past. The photocurrent effect observed by an individual Er atom has been reported at 4 K, therefore, far from practical operation temperatures [3]. There, the ^4^$I_{13/2}$→^4^$I_{15/2}$ transition of the ${\mathrm{Er}}^{3+}$ ion in the silicon transistor is stimulated by a laser tuned at the resonance wavelength. We already explored single atom and impurity band effects in single ion implanted transistors [10,11,12,13] by including As [14], P [15] and Ge [16] atoms in view of nanoelectronic application, and the individual photon emission regime by photoluminescence of ErO*_x_* dots in silicon [6]. Several near-infrared detectors have been demonstrated in the past starting from Reference [17], including some based on CMOS technology with high sensitivity [18,19], but they are not frequency-selective, which would be relevant to reduce dark counts without the use of filters when detection of single monochromatic photons is targeted in an integrated device. In this article, the photocurrent of an ErO*_x_* doped transistor is modulated by telecom wavelength irradiation as a function of both irradiation power and frequency. We investigate the room temperature photocurrent regime, where applications can be designed, by exploiting a relatively small number of Er atoms, of the order of $4\times 10^{4}$, fed by a laser operated in the μW regime. In the following, the process to co-implant Er and O in back-gated transistors is outlined, and the photocurrent characterization is described at telecom wavelengths around 1550 nm. The photocurrent is proportional to the power of the laser and it reveals an absorption frequency dependence at coincidence with the Er absorption line. ErO*_x_* co-doped transistor can operate at room temperature as a frequency selective light sensor, tuned at the same wavelength range of Er emission, towards nearby room temperature single photon emitter [20] and receiver [21] resonators based on few Er atoms.

## 2. Materials and Methods

### 2.1. Device Fabrication and Transport Characterization

**Figure 1:**
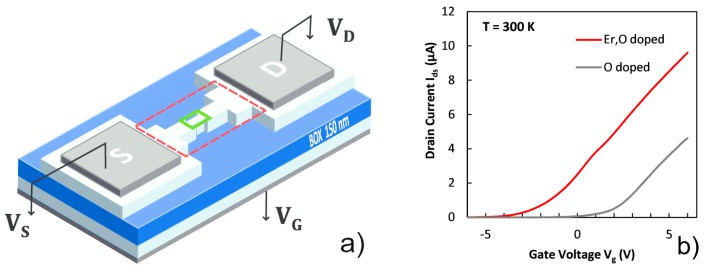
(**a**) Schematic of a transistor fabricated in an n-type (100) silicon on insulator (SOI) wafer. The transistor is based on a phosphorous-doped ($1\times 10^{21}\;{\mathrm{cm}}^{-3}$) $n^{+}$ source and drain regions and a phosphorous-doped ($1\times 10^{15}\;{\mathrm{cm}}^{-3}$) $n^{-}$ channel region (size of L = 1 μm, W = 1 μm) and operates in the accumulation mode. The red dashed line encloses the optically investigated area. (**b**) I-V curve of two transistors differing only by the Er implantation measured at room temperature with $V_{DS}=100$ mV. The shift of the threshold voltage is caused by the defect formation in the silicon band gap after 900 °C annealing.

Back-gated transistors were fabricated on a silicon-on-insulator (SOI) wafer by conventional complementary metal-oxide-silicon (CMOS) processes. The $n^{+}$ source and drain regions were doped with phosphorous at a concentration of $10^{21}$ cm^−3^, and the $n^{-}$ channel region was left undoped with $10^{15}$ cm^−3^, thus, the transistors operate in the accumulation mode. Each transistor consists of 150 nm-buried oxide layer and 90 nm-SOI layer capped with 15 nm-silicon dioxide layer. The transistor size ranges between *W* = 1–10 μm, with L=1 μm. The photocurrent effects reported in this work refer to the minimum size of W = 1 μm. The channel was doped with erbium at a dose of $3\times 10^{13}$ cm^−2^ at 20 keV. Oxygen was then implanted at a dose of $2.5\times 10^{13}$ cm^−2^ at 25 keV with a sacrificial layer deposited on the wafer to adjust the implantation depth of the two dopants. After removing the sacrificial layer, the wafer was thermally annealed at 900 °C for 30 min to promote the association of erbium and oxygen. Because of the out-diffusion of Er from the surface consequent to the annealing process, which reduces the Er of a factor of ≈7 [6,22], we estimate that the Er atoms in the channel of the transistor are of the order of $4\times 10^{4}$. According to the literature, the annealing of Si:ErO*_x_* at 900 °C determines the formation of a defect which lies at $E_{C}\approx -150$ meV where $E_{C}$ is the conduction band edge, and it is partially ionized by thermal excitations at 300 K [8]. Such defect is dominant at high annealing temperature with respect to other Er-O related defects, while other Er defects disappear when O is present [23]. Such defects are not electrically explored in diode structures, while they can be explored by a transistor device, thanks to the control by the back gate voltage (Figure 1a). The formation of such defects is observed by the sub-threshold transport of the devices. Compared to the transfer characteristics curves of a control transistor not implanted with Er (Figure 1b), the Er-related defects at −150 meV determine a negative shift of the threshold voltage (data refer to L = 1 μm, W = 2 μm devices at $V_{DS}=100$ mV). During a voltage sweep from negative to positive values the first accessed energy levels are those provided by the ErO defects, and only at about 150 meV higher in energy the conduction band is directly accessed.

### 2.2. Optical Setup for Photocurrent Characterization

**Figure 2:**
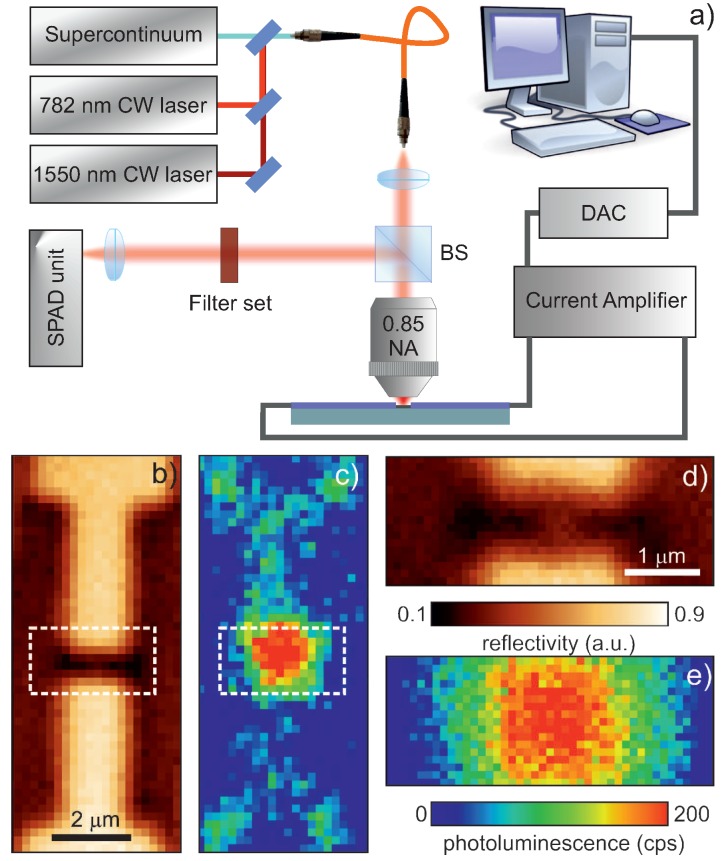
(**a**) Schematic of the setup used for the optical and electrical characterization of our sample. BS: beam splitter cube, DAC: data acquisition card. (**b**) Reflectivity and (**c**) photoluminescence (PL) maps of the whole transistor recorded at the telecom band while exciting it with the continuous wave (CW) laser at 782 nm. (**d**,**e**) Zooms of the maps shown in (**b**,**c**), respectively (see dashed lines), which correspond to the active region of the transistor. The PL is mostly coming from the area of the Er-implanted channel.

The setup employed for the optical and photocurrent characterization is the modified commercial confocal microscope (WITec GmbH, Ulm, Germany) sketched in Figure 2a. The photoluminescence (PL) of the sample is excited by a continuous wave (CW) fiber-coupled laser diode at 782 nm wavelength. Light from such laser source, collimated and reflected by a beam splitter cube (Thorlabs Inc., Newton, NJ, USA), is focused by a 0.85 NA objective (Nikon Instruments Europe BV, Amsterdam, The Netherlands) to a diffraction-limited spot of about 1 μm in diameter on the sample. The emission from Er ions is collected through the same objective in epireflection geometry, and it is sent to a set of long-pass filters (1300 and 1350 nm cut-off wavelengths, Thorlabs Inc.) to reject any residual reflected pump light for the PL detection. The filtered emission is then imaged with an InP/InGaAs single photon avalanche diode (SPAD). To excite the photocurrent in the device, instead, we switch our light source to a CW fiber-coupled laser diode with a wavelength of 1550 nm.

## 3. Results and Discussion

### 3.1. Photoluminescence Characterization

We now turn to the photonic characterization of the device. First, we performed PL and photocurrent measurements with the modified commercial confocal microscope introduced in the previous section and sketched in Figure 2a. We record emission PL and reflectivity maps form areas up to 80×80 μm^2^ from the sample, by raster scanning it through the excitation and collection process with a piezoelectric stage. Figure 2b,c shows the reflectivity and emission maps respectively recorded from our Er-doped transistor. From a comparison of the sample topography and PL maps, we can confirm that the majority of the emission takes place in the channel area (highlighted region in Figure 2c and its zoom in Figure 2e), thus, verifying that the Er ions implantation is confined to this region. The lower intensity signal coming from the adjacent regions is attributed to a small PL contribution from the transistor contacts, while emission from the substrate in the spectral region is considered to be negligible.

### 3.2. Photocurrent Characterization

**Figure 3:**
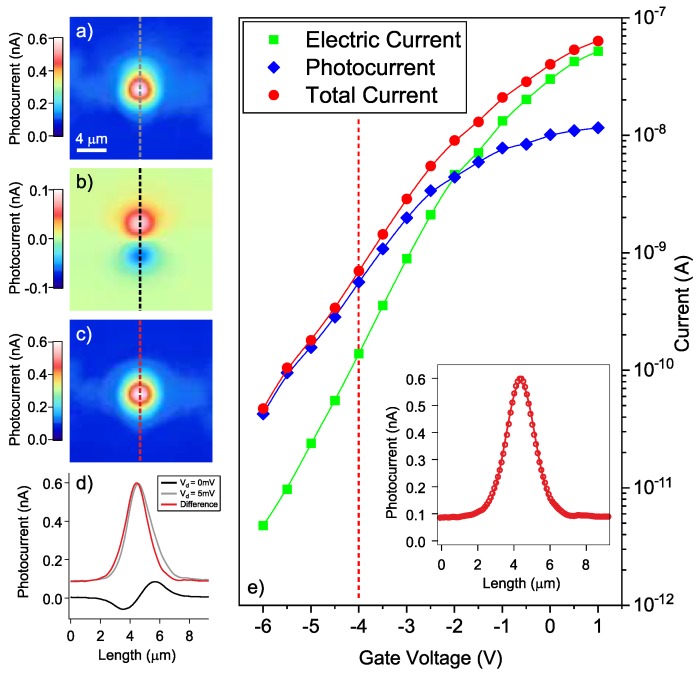
(**a**) Overall photocurrent map recorded on the transistor when exciting the sample at 1550 nm while applying $V_{G}$ = −4 V and $V_{DS}$ = 5 mV. (**b**) Photocurrent recorded when no net VDS is applied. (**c**) Pure photocurrent map obtained by subtracting the overall photocurrent in (**a**) by the background photocurrent in (**b**). (**d**) Vertical line profiles drawn on (**a**–**c**). (**e**) Drain photocurrent in ohmic regime ($V_{DS}$ = 5 mV) as a function of the gate voltage in dark condition (no light, green squares) and the peak photocurrent recorded on maps such as (B) while illuminating the device with an optical power of about 1 mW (red circles). The photocurrent (blue diamonds) calculated by taking the difference between the current in ON (signal) and OFF (back-ground) conditions, i.e. the spots where the photocurrent was recorded while photoexciting on the substrate and on the Er-doped channel, respectively.

To perform the photocurrent characterization, we switch to a laser source with a wavelength of 1550 nm, and we couple it to the sample through the same setup described above. The drain-source current of the ErO*_x_* doped transistor is converted into a voltage signal using a custom transimpedance amplifier with a conversion factor of 10 nA/V and a bandwidth of 16 Hz. The output voltage of the amplifier is connected to the input channel of the microscope and converted to the digital domain with a resolution of 3 pA simultaneously to the optical signal (see Figure 2a). In this way, optical reflectivity and photocurrent maps are simultaneously recorded, as exemplified in Figure 3a,b respectively. By comparing the sample topography and the photocurrent maps, the main current contribution originates when the Er-implanted channel region is optically excited (red dot in Figure 3b, ON state), while some background electrical current is generated from the other areas of the sample (green dot in Figure 3b, OFF state). By subtracting this background current signal recorded on the substrate to the current coming from the optical excitation of the transistor channel, we can extract the pure photocurrent generated with a $V_{DS}$ = 5 mV by telecom wavelength irradiation, as plotted in Figure 3c (blue diamonds). Here, the two previously mentioned regimes, namely the photogenerated current and the conduction band transport respectively, are distinguishable. In particular, the photocurrent generated for $V_{G}≲-2$ V is the one related to the presence of the defects.

It is worth mentioning here that by performing measurements with a non-zero gate voltage applied and zero bias between source and drain, we could observe a spurious current signal with two opposite signs at coincidence with the electrode edges, as shown in Figure 3b. Moreover, we observe that when the channel is almost completely depleted at gate voltages $V_{G}$ below −6 V, a presumably different effect induces a photocurrent of the order of pA with reversed sign at the source and the drain sides respectively. This artifact has been already reported in the literature [24] and it is attributed to trapped photo-generated electrons that are effective in the suppression of the electric field near the electrodes. Although the rejection of such spurious signal is rather challenging, we were able to avoid such an artifact by collecting the signal in the middle of the channel (see red spot in Figure 3b), where the signal level for the photocurrent induced by the trapped electrons at the electrodes is negligible. A more detailed characterization of such effect is reported in the Figure S1 in the Supplementary Material.

**Figure 4:**
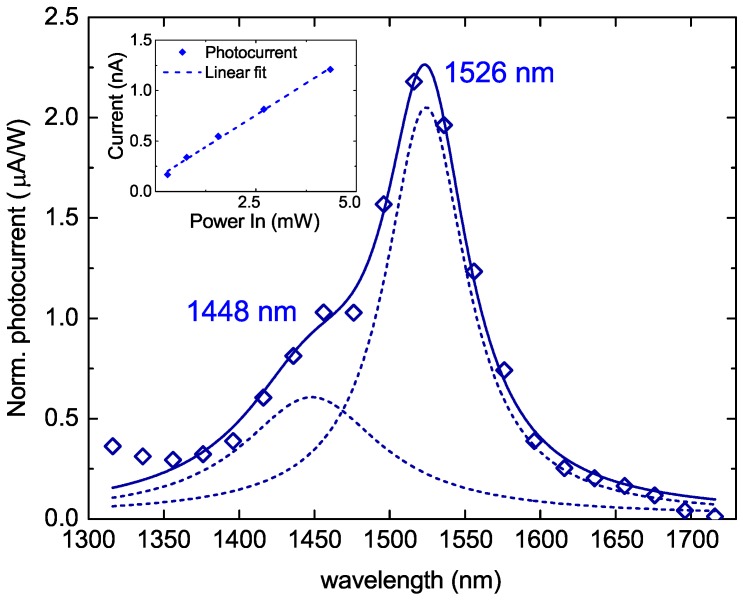
Frequency dependent photoconductivity measurements observed from the photocurrent, realized by exciting the Er-doped transistor with a supercontinuum light source, normalized by the optical power throughput of the source through our setup (empty diamonds). Double peak Gaussian fit to highlight the position of the spectral features of the photoconductivity measurement (dashed lines), which reproduce the wavelength dependent absorption cross-section of Erbium [26]. Inset: linear dependence of the photoconductivity on the input optical power, measured at λ = 1550 nm.

We then performed wavelength dependent photocurrent measurements using the same acquisition setup mentioned above, by switching to a supercontinuum light source. In order to observe effects related to the defects only, the gate voltage is set to −4 V (see dashed red line in Figure 3c), at which value the defects are partially filled while the conduction band is almost empty at room temperature. To separate the different excitation wavelengths within the wide band of the exciting supercontinuum source, the light was chromatically dispersed by a Pellin-Broca prism and then selected by a slit before being coupled to a single mode fiber. In this way we could attain a relatively broadband monochromator, which allowed us to vary the wavelength of the excitation between 1400 nm and 1700 nm in steps of 20 nm with an input power level of about 1 mW for each spectral window. This experimental arrangement is the exact reciprocal of that described in detail elsewhere [25]. We also determined the optical throughput of our system by measuring the laser power impinging on the sample at each operating wavelength, as a normalization reference for the photocurrent measurement. The pure photocurrent generated by the Er-doped transistor is extrapolated from the maps recorded for each exciting wavelength, as described above. The photocurrent spectrum thus, obtained is then normalized by the power spectrum of the supercontinuum source through the optical setup to account for the achromatism of the setup. The result of this operation is shown in Figure 4a. The normalized photocurrent spectrum thus, obtained shows a main peak around 1526 nm, with a shoulder around 1448 nm. Such spectral features are in excellent agreement with the wavelength dependent absorption cross-section of commercial Er-doped optical fibers. Additionally, as shown by the linearity of the photocurrent dependence on the optical pump power at λ = 1550 nm (see the inset of Figure 4), the Er-doped transistor is operating far from its electrical saturation regime.

## 4. Conclusions

To conclude, we demonstrated that an ErO*_x_* co-doped silicon transistor exhibits a tunable response at room temperature according to a linewidth profile typical of erbium absorption. Electrons are pumped from the ErO*_x_* complex activated by the annealing at 900 °C. The linear dependence of the signal over the impinging power demonstrates that the Er-doped transistor is operating far from electrical saturation. The relatively small number of photoexcited atoms estimated (≈$4\times 10^{4}$), combined with a frequency-selective response, points towards a viable employment of ErO*_x_* defects in resonator-based single photon detectors at telecom wavelength at room temperature.

## Supplementary Materials

The Supplementary Materials are available online at https://www.mdpi.com/2079-4991/9/3/416/s1.
