# Supplementary material for: Room Temperature Resonant Photocurrent in an Erbium Low-Doped Silicon Transistor at Telecom Wavelength
Source: Nanomaterials (Basel). 2019 Mar 11;9(3):416. doi: 10.3390/nano9030416 (PMC6474141; doi:10.3390/nano9030416)
Supplement: Supplementary file 1 [file nanomaterials-09-00416-s001.pdf]

Supplementary Information

Room temperature resonant photocurrent in an erbium low-doped silicon transistor at telecom wavelength

M. Celebrano[1], L. Ghirardini[1], M. Finazzi[1], G. Ferrari[2], Y. Chiba[3], A. Abdelghafar[3], M. Yano[3], T. Shinada[4], T. Tanii[3], and E. Prati[5]

[1]Dipartimento di Fisica, Politecnico di Milano, Milano, Italy [1]DEIB, Politecnico di Milano, Milano, Italy [3]School of Science and Engineering, Waseda University, Tokyo, Japan [4]Center for Innovative Integrated Electronic Systems, Tohoku University, Sendai, Japan; [5]Istituto di Fotonica e Nanotecnologie, Consiglio Nazionale delle Ricerche, Milano, Italy, email: enrico.prat@cnr.it

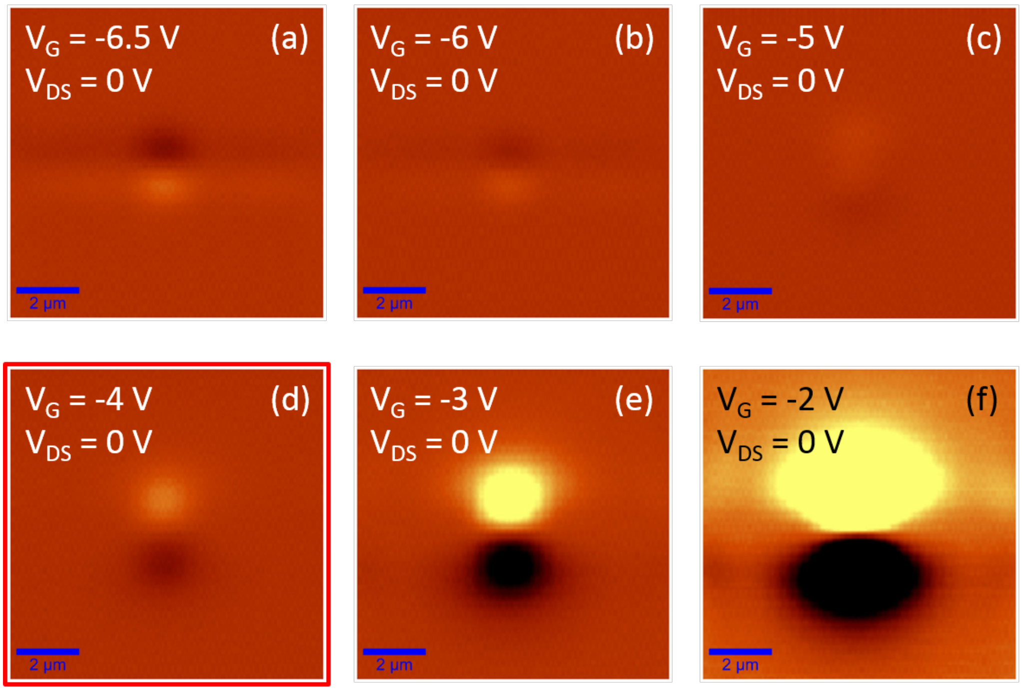

Fig. S1. Photocurrent maps recorded at an excitation laser wavelength of about 1550 nm and 1 mW power. Electrical parameters are V_{DS} = 0 mV and V_G = -6.5 V (a), -6 V (b), -5 V (c), -4 V (d), -3 V (e) and -2 V (f), respectively. The minimum (maximum) photocurrent corresponds to -0.2 nA (+0.2 nA) in panel (f). The vertical axis corresponds to the direction of L of the transistor, while the horizontal axis to W. Such measurements provide the background photocurrent induced in the transistor when no bias is applied between the source and the drain. A non –vanishing photocurrent with two opposite signs is excited at both the source and the drain junctions [1] so the two contributions cancel at the center of the device. The characterization of the effects reported in the main text have been performed where the two contribution cancel. We observe that when the channel is almost completely depleted at gate voltage V_g below -6 V, a presumably different effect induces a photocurrent of the order of pA with reversed sign at the source and the drain sides respectively.

[1] T. Agostinelli, M. Caironi, D. Natali, M. Sampietro, P. Biagioni, M. Finazzi, and L. Duò, J. Appl. Phys. 101, 114504 (2007).
